# Experiences of patients and healthcare professionals of NHS cardiovascular health checks: a qualitative study

**DOI:** 10.1093/pubmed/fdv121

**Published:** 2016-10-17

**Authors:** R. Riley, N. Coghill, A. Montgomery, G. Feder, J. Horwood

**Affiliations:** 1 School of Social and Community Medicine, University of Bristol, BristolBS8 2PS, UK; 2 Faculty of Medicine & Health Sciences, University of Nottingham, Nottingham NG7 2UH, UK

**Keywords:** population-based and preventative services, primary care, public health

## Abstract

**Background:**

NHS Health Checks are a national cardiovascular risk assessment and management programme in England and Wales. We examined the experiences of patients attending and healthcare professionals (HCPs) conducting NHS Health Checks.

**Methods:**

Interviews were conducted with a purposive sample of 28 patients and 16 HCPs recruited from eight general practices across a range of socio-economic localities. Interviews were audio recorded, transcribed, anonymized and analysed thematically.

**Results:**

Patients were motivated to attend an NHS Health Check because of health beliefs, the perceived value of the programme, a family history of cardiovascular and other diseases and expectations of receiving a general health assessment. Some patients reported benefits including reassurance and reinforcement of healthy lifestyles. Others experienced confusion and frustration about how results and advice were communicated, some having a poor understanding of the implications of their results. HCPs raised concerns about the skill set of some staff to competently communicate risk and lifestyle information.

**Conclusions:**

To improve the satisfaction of patients attending and improve facilitation of lifestyle change, HCPs conducting the NHS Health Checks require sufficient training to equip them with appropriate skills and knowledge to deliver the service effectively.

## Background

Cardiovascular disease (CVD) is one of the leading causes of premature mortality and morbidity in the UK^1^ with an estimated cost to the NHS of £14.4 billion.^2^ The incidence of CVD is projected to rise due to an ageing population and a higher incidence of hypertension and Type 2 diabetes, linked to obesity.^1^ NHS Health Checks were introduced in England in 2009 as part of a government commitment to tackle avoidable deaths, disability and reduce health inequalities in England associated with the above.^1,3^

NHS Health Checks are a national cardiovascular risk assessment and management programme for people aged 40–74 years who do not have existing CVD. Local authority public health departments have commissioning responsibilities for NHS Health Checks; these are predominantly conducted in primary care, by nurses and healthcare assistants (HCAs). They aim to identify patients at-risk of developing heart disease, stroke, diabetes and kidney disease. Patients receive a QRisk2 score^4^ which provides a percentage estimate of their 10-year risk of developing CVD. The QRisk2 algorithm is based on cardiovascular risk factors, including family history, socio-economic status and ethnicity.^4^ Patients at increased risk of CVD (>10%) are offered behavioural interventions such as referral to local lifestyle modification services (e.g. smoking cessation and healthy eating support).^5^ Those whose tests suggest chronic conditions such as hypertension, chronic kidney disease or hypertension are referred to their general practitioner for formal diagnosis and treatment.

Initial evidence suggests that some patients value NHS Health Checks because they provide reassurance, reinforce pre-existing healthy lifestyles and prompt patients to make healthy lifestyle changes.^6^ Patients also value the longer appointment and opportunity to talk to a healthcare professional (HCP).^7,8^ However, there are conflicting findings about the overall value of NHS Health Checks and their potential to reduce health inequalities and cardiovascular events.^9–14^ A recent study, found there were no differences in the reported prevalence of diabetes, hypertension, coronary heart disease and chronic kidney disease, in practices which offered NHS Health Checks compared with practices who provided usual care.^15^ The variability and effectiveness of approaches employed to promote lifestyle change has also raised concerns about the value of NHS Health Checks.^6,16^ Whilst limited research has examined patients' experiences of attending an NHS Health Check,^6,16,17^ there is a paucity of research exploring the implications for patients attending and the views of HCPs delivering the NHS Health Checks. This study aimed to improve our understanding of NHS Health Checks by investigating the experiences and views of both patients attending and HCPs involved in implementing NHS Health Checks. Examining the views and experiences of both HCPs and patients enables the identification of factors that may enhance our understanding of how best to deliver NHS Health Checks and consider the implications for policy and practice.

## Methods

Semi-structured interviews were conducted with patients who had attended an NHS Health Check and with HCP, who had delivered NHS Health Checks; this included General Practitioners (GPs), practice nurses, HCAs and pharmacists. Eight primary care practices in Bristol were purposively selected to access populations from a range of socio-economic backgrounds (SES) using practice level indices of multiple deprivation (IMD) scores.^18^ Practices in the most deprived quintile are designated as ‘1 SES’ and practices in the most affluent quintile are categorized as ‘5 SES’. Eligible patients were identified through a search of patient records for (i) patients who had undertaken a health check within the previous 6 months and (ii) patients with low (<10%), medium (>10% to <20%) and high (>20%) QRisk2 scores. A total of 541 invitations were sent to patients and 95 (14%) replied.

For those who agreed to be contacted, a purposive sample was recruited accounting for SES, QRisk2 scores, ethnicity, gender and age. Face-to-face (*n* = 22) and telephone (*n* = 6) interviews were conducted between April 2013 and February 2014. Patients were informed that the interview would explore their attitudes and expectations of the NHS Health Check, their experience of the process, receipt of results and information provided. Interviews with patients were conducted between 1 and 6 months after they received their NHS Health Check. Patient face-to-face interviews took place in the patient's home.

HCPs were recruited via invitation letter and purposively sampled from the same eight practices as well as from an additional three practices, across a range of SESs. Eighteen HCPs were invited, 15 accepted and 3 did not respond. All face-to-face interviews were conducted in the workplace. HCPs were informed that the interview would explore their views on the purpose and value of NHS Health Checks, and their experiences of delivering the Health Check, including communication of the results. All interviews were conducted by one researcher (R.R.) and lasted between 20 and 60 min. This study was approved by the NHS Ethics Committee South West 4 (ref 10/H0102/39). During all interviews, a flexible topic guide was used to ensure key issues were covered, but allowing participants to introduce unanticipated issues (see Box 1).

Box 1 **Interview topics for healthcare practitioner and patient interviews**

**Interview topics**

Healthcare Practitioners

Understanding of NHS Health Check—Aims, value, role.

Usual practice pre NHS Health Check—Conducting health checks, how were patients identified/managed.

Implementation of NHS Health Checks—Method, rationale for invite, uptake, challenges, training.

Conducting NHS Health Checks—How are checks carried out and skills required, experiences of communicating the results and information and dealing with referrals and follow-up.

Views of the process—Benefits, weaknesses, training needs.

Patients

Pre-health check—Patients feelings about their health in general, how were patients informed and what information did they receive, understanding of the purpose and motivations for attending, feelings/thoughts about attending, perceived benefits/disadvantages.

Experience of health check—Who carried out the check, experience of check and results, understanding of results and information, benefits/disadvantages of check.

Post-health check—What happened next, i.e. referrals, lifestyle changes.

Data collection and analysis were conducted in parallel and interviews continued until data saturation was reached and no new themes were arising from the data.^19^ With written informed consent, interviews were audio recorded. Anonymized transcripts were imported into NVivo10 and analysed using a thematic analysis.^20^ Analysis was ongoing and iterative, informing further data collection. R.R. initially coded the data and a subset of six transcripts were independently analysed by J.H. to contribute to the generation and refinement of codes and thematic categories to maximize rigour. Professional and patient interviews were analysed separately with data triangulation of emerging themes.

## Results

### Sample characteristics

A total of 28 patients interviewed (see Tables 1 and 2 for participant characteristics). A significant proportion of patients who participated were <60 years. This may be accounted for by the purposive sampling of patients with a medium and high-risk score. The age of the patient is incorporated into the QRisk2 algorithm; the higher the age, the higher the risk. The analysis identified three key themes which are described below with the use of verbatim quotes. All names refer to pseudonyms.

**Table 1:** Patient demographic characteristics (*n* = 28)

*Characteristic*	**n**
Age (years)	
40–44	1
45–49	1
50–54	2
55–59	3
60–64	9
65–69	6
70+	6
Ethnicity	
White British	23
White other	2
Black	2
Asian	1
Gender	
Female	16
Male	12
Socio-economic status^a^	
1 (most deprived)	11
2	6
3	6
4	4
5 (most affluent)	1
Cardiovascular QRisk2 score	
High (>20%)	11
Medium (>10% to <20%)	11
Low (<10%)	6

^a^Measured as IMD quintile from home postcode.

**Table 2:** Healthcare professional's demographic characteristics (*n* = 15)

*Characteristic*	**n**
Job title	
GP	5
Practice nurse	5
Pharmacist	1
Healthcare assistant	3
Pharmacist	1
Age (years)	
25–34	1
35–44	3
45–54	8
55–64	3
Race/ethnicity	
White British	11
White other	3
Black	1
Gender	
Female	10
Male	5
Socio-economic status^a^	
1 (most deprived)	6
2	3
3	3
4	0
5 (most affluent)	3

^a^Measured as IMD quintile from practice postcode.

### Motivations for attending a health check

Patients' reasons for attending an NHS Health Check were varied. However, the most common motivations, across all socio-economic groups, were underpinned by health beliefs associated with the value of NHS Health Checks as a preventative programme, a desire to be informed about risk factors or early warning signs of CVD and values attached to positive lifestyle changes:

you do know if there's something that you should be concerned about … giving you options to sort of deal with things like that, and diet, you know. (Mrs Pope, 50–54 years, SES 1, low risk)

if they pick up something … you're sort of doing preventative rather than actually dealing with something which … may be worse (Mrs Monroe, 65–69 years, SES 3, medium risk)

Some patients were motivated to attend because they valued preventative programmes as informed by a familial experience of CVD and other diseases:

We have got heart problems in our family … so this is why I do go for regular check-ups’ (Mrs Henderson, 55–59 years, SES 1, medium risk)

it can pick up things in time. I mean my grandmother had cervical cancer and didn't get it treated (Mrs Forde, 55–59 years, SES 1, low risk)

Several patients viewed the NHS Health Checks as a more general health check rather than specifically targeting CVDs:

I would hope that it [NHS Health Check] threw up anything I needed to bother about, or other people needed to bother about … heart trouble, chest trouble, forms of cancer, diabetes. (Mr Taylor, 70+ years, SES 5, high risk)

I think it's [NHS Health Check] a good idea … Well high blood pressure is one, usually they can pick up anything on the heart that is not quite normal, your lungs. … all that kind of thing you know. How you move, you know joints. (Mrs Flint, 70+ years, SES 1, medium risk)

A minor theme identified that a few patients were motivated to attend an NHS Health Check for proactive and preventative reasons as they did not wish to be a burden on their family, the NHS or society:

I try and keep myself as healthy as I can, to try and reduce that kind of pressure on society and not put too much strain on … sort of family, friends, and NHS (Mr Adams, 65–69 years, SES 2, low risk)

I try to be as healthy as I can, to try and reduce that kind of pressure on society (Mrs Kelly, 60–64 years, SES 4, low risk)

A small minority of participants indicated that they took up a health check because they had more time now they were retired:

… because I'm retired now, I actually have time to sit down and think about these things (Health Check invite) … I didn't really have the time … or I didn't want to give time or space (Mr Adams, 65–69 years, SES 2, low risk)

HCPs, predominantly GPs and nurses, who consulted with patients across a range of socio-economic groups, concurred with these motivations. They reported that a high proportion of patients attending for a Health Check were proactive and had healthy lifestyles—a patient group referred to as the ‘worried well’:

a lot of the ones that came in were … well, were perfectly healthy but just wanted to get everything checked out. (ID 1, nurse, SES 1)

I think you'll always get the worried well in any setting for a lot of this … actually these are the people who don't smoke, drink modestly, take lots of exercise, are thin; you know, I would call them well behaved patients. And for them it was just reassuring. (ID 15, nurse, SES 1).

As a result of the perceived higher uptake from healthier patients, some HCPs, particularly GPs and nurses, voiced concerns about whether the current approach for identifying patients represented an efficient and cost-effective method of screening for cardiovascular risk in the long-term:

I don't think there is an awful lot of value. I think you'll pick up a few people a little bit earlier. Now whether that's worth the cost, obviously it's great for those individual patients, whether that's worth the cost of running a programme like this. I'd be amazed if it was. (ID 6, nurse, SES 1)

Some HCPs, specifically GPs and nurses, raised concerns that the patients who would benefit the most were the ones who were least likely to attend:

if you send out an invite to a large number of people then the people who present themselves (laughs) er might well fit into that worried well category, um won't necessarily be um the HGV driver who works long hours and smokes a lot (ID 7, nurse, SES 2)

### Communicating results and lifestyle advice

HCPs and patients across SES, with varying risk levels reported that communicating cardiovascular risk and accompanying, personalized lifestyle information is important, and that failure to do so can be dissatisfying, frustrating and confusing for patients. These accounts indicate that patients are not consistently offered, or recall receiving, detailed personalized information and lifestyle advice to support them in making changes:

my cholesterol is high … and, I had a score saying sixteen per cent diabetes in ten years. What does that mean? I've got no idea what that means. It sounds bad because it's higher than it's meant to be but is it? And it was that kind of information which was the kind of the bit beyond, you know, eat less, exercise more, don't smoke, don't drink … that would have been useful that didn't really seem to be part of what was on offer there … So, I think the kind of advice that was on offer was actually very, um, simple (Mrs Jackson, 60–64 years, *SES 1*, low risk)

she [practice nurse] talked about having a reasonable amount of exercise, but it was more questions than answers, to be honest. I don't remember much, any advice, specifically. I remember questions about what I did being asked, or what I didn't do, but I don't remember much advice. (Mr Taylor, 70+ years, SES 5, high risk)

Some patients were left feeling uncertain about the significance of their results and expressed a desire to be more informed about the implications of their results by an HCP who had the required skill set and knowledge:

I understand it [cholesterol result] is now on the high side of the normal. Whatever that means … because it was only a receptionist that told me and … she said there's no worries and I thought I'm not sure she's just telling me this … So it would have been helpful to have spoken to maybe a nurse or … you know, a word from the GP would have been helpful. (Mrs Monroe, 65–69 years, *SES 3*, medium risk)

Some high-risk patients had not understood the significance of their numerical QRisk2 score, or implications of any identified risk factors for example, raised blood pressure. These patients assumed that they were healthy and were reassured by their NHS Health Check, despite being categorized as having a high risk for CVD:

although I know that I'm healthy, it's nice to be told by somebody who's a bit in the job (Mr Nicholas, 60–64 years, SES 1, high risk)

I'm thinking, “Well I'll just keep plodding on and plodding on.” I don't know whether high blood pressure does you any harm over a long term or whether it's, you know, as long as it's not very, very high. (Mr Ustinov, 60–64 years, SES 1, high risk)

It says if your risk is high you will be given advice about how to lower your risk. You may be given medicines or offered further support to help reduce your risk. … I presume 20.8 [QRiskscore2] or whatever is low … Well I suppose it is. Um (pause) uh, I suppose it's reassuring to know that my levels are fine (Mrs Brown, 65–69 years, *SES 2*, high risk)

This last patient assumed that her high QRisk2 score of 20.8% was low, yet this is the threshold at which a range of preventative pharmacological and behavioural interventions would be offered.

Concerns were raised about whether some HCPs, particularly HCAs, are equipped with the skills and knowledge to provide a more informed and patient-centred consultation. This is in contrast to the more task focused consultation, which concentrates on the biomedical components of the NHS Health Check. This approach may not consider, or may overlook the patient's wider psychosocial needs or concerns:

My expectation of a healthcare assistant is that they are using just strictly task oriented procedures, and not really dealing with the whole person picture … that's an economic decision … that it costs more to have me do that review than to have a healthcare assistant do that review (ID 7, nurse, SES 1)

Other HCPs, particularly nurses and GPs, raised concerns about whether some staff had the skill set to communicate risk information effectively, to patients:

I would wonder how well some of the other girls [nurses and HCAs] here are about giving what CVD risk means, how you translate that into patient friendly sort of information, how you sort of like um talk about, you know, your risk of a cardiovascular event over however much time. (ID6, nurse, SES 1)

In contrast, HCAs were largely positive and confident in their role, and felt that they had considerable transferable skills such as taking blood pressure, monitoring risk and making referrals when appropriate:

the stuff that I'm doing for the health check is what I do in my everyday job anyway, so I'd actually say I'm clued up on what I'm doing … ‘cos if I've got a patient come in and they've got high blood pressure, we will do the same as we do for the health check…we'll check it again to make sure. If it's still high we will do an ECG, take bloods, and then tell them to go back and see the doctor to get their result, and the doctor takes it further (HCA, ID 2, SES 1)

### Implications of attending an NHS Health Check

This theme highlighted the differences in the psychological and behavioural impact of attending for an NHS Health Check and consisted of three sub-themes relating to: (i) reassurance, reinforcement and relief, (ii) anxiety provoking and (iii) behaviour change.

#### Reassurance, reinforcement and relief

For many patients who received low and medium QRisk2 scores, NHS Health Checks served to reassure and reinforce healthy lifestyle behaviours and for some the results were a relief:

going through various sort of checks … like cholesterol … and talking about my diet, it … reinforced and made me think … I'm actually doing all the things I probably should be doing. (Mrs Pope, 50–54 years, SES 1, low risk)

probably because it was a clean bill of health … and … ‘phew! [Laughs] Thank god for that.’ And then I walked out and sort of punched the air … it felt really good. (Mr Adams, 65–69 years, SES 1, medium risk)

#### Anxiety provoking

For some patients who attended a health check, the experience caused anxiety after receiving unexpected results:

it was quite scary actually to think oh, I've got something wrong with me after all … as I say I went into this whole thing kind of, kind of blithely thinking that I'm in good health. And I think it is probably quite a sort of salutary lesson that you might not be. (Mrs Jackson, 60–64 years, SES 1, low risk)

Some patients also experienced additional anxiety whilst waiting a re-test or follow-up tests:

because I had high blood sugar … that did cause me a bit of anxiety … so I went back for a fasting blood test … and then I went back a couple of weeks later … . And that was clear. That was okay … So that was a bit of a relief. I don't really want to have diabetes. (Mr Gray, 55–59 years, SES 3, medium risk)

HCPs, mainly GPs and nurses, recognized the potential for NHS Health Checks to raise concerns amongst patients or compound pre-existing anxiety:

sometimes some people are in shock really, especially when it comes to the cholesterol, they think, “Oh my God, I didn't know it was that high,” (ID 2, healthcare assistant, SES 1).

it [NHS Health Check] can make people feel bad or worried or more anxious … I see a lot of anxious people, in my opinion. They've got lots of life stresses so they're naturally anxious, and I make them more anxious (ID 16, GP, SES 2)

#### Behaviour change

Attending an NHS Health Check and being informed about cardiovascular risk factors also prompted some patients, particularly medium- and high-risk patients, to make healthy lifestyle changes:

I did make a concerted effort and … did lose a bit more weight … because coupled with like high cholesterol and blood pressure, I thought oh a dodgy combination. (Mrs Monroe, 65–69 years, *SES 3*, medium risk)

I've changed my diet um and, and lost a stone in weight I think as a result actually. So I'm quite happy with that, that makes me feel even healthier (Mr Gray, 55–59 years, *SES 3*, medium risk)

For some patients, attending an NHS Health Check prompted lifestyle changes and encouraged patients to talk more about their health:

I was avoiding salts and sugar … I do a bit more than I was doing, you know, the exercises … , I keep an eye on my weight … Um and I suppose we talk about it a bit more than – than I used to … And my partner … is fairly conscious about it, and she talks quite a lot about it, … “I've been on the scales,” you know, when she goes to the gym. (Mr Raj, 60–64 years, SES 1, high risk)

Although some patients reported making positive lifestyle changes, others resisted change or were less prepared to engage with behavioural interventions, e.g. smoking cessations clinics, when offered:

I was told that there was a smoking cessation clinic there. I think she [nurse] could tell that if I was going to do that I will do it, but I wasn't going to do it, no, no, no. (Mrs Cookson, 50–54 years, SES 2, medium risk)

Some patients, including Mrs Cookson, felt that they would prefer to give up smoking on their own accord rather than with the support of an HCP.

A minority of patients understood the implications of their results and recognized the value of making positive lifestyle changes, yet felt constrained by psychosocial circumstances, e.g. bereavement, stress or socio-economic barriers, such as shift work:

I did give it [smoking] up and then I lost my mum and obviously I went back to smoking … If I'm going to do it [quit smoking] I'm going to do it myself. I haven't had a fag for two days now but it's when I go to work because my job is so stressed … I know you shouldn't use a cigarette for an excuse but it's like I've got to have a fag. (Mrs Bonney, 60–64 years, SES 1, high risk)

And in all honesty, I'd love to lose 2 stone … I'm 60 – it's just having the energy after doing an 8–10 hour shift to want to do something, that's the problem … But shift workers can't … it's immensely disruptive.’ (Mr Ustinov, 60–64 years, SES 1, high risk)

## Discussion

### Main findings

Patients were motivated to attend an NHS Health Check due to their health beliefs, the perceived value of the programme, family history of cardiovascular and other diseases and expectations of receiving a general health assessment. HCPs raised concerns about the potential for inequity in uptake and the effectiveness of the programme. Patients indicated that they do not always feel well informed about the implications of their results and did not always receive detailed and personalized lifestyle information or advice to accompany these results. This was supported by HCPs who had concerns about the skill set of some HCPs to communicate risk and lifestyle information effectively. The reported experiences of some patients highlight that some individuals who were given a high QRisk2 score had not fully understood its significance. Perceived benefits of the check included reassurance, relief and reinforcement of healthy lifestyles with some patients making positive lifestyle changes. Some patients identified psychosocial barriers to lifestyle change or experienced anxiety from unexpected results or whilst waiting follow-up tests.

### What is already known about this topic

Few studies have reported the experiences and views of patients and HCPs regarding NHS Health Checks.^6,16,21^ Previous findings report that NHS Health Checks are commonly viewed as a more general screening opportunity by patients.^6,17^ Factors affecting attendance for an NHS Health Check included: individuals’ perceived susceptibility to cardiovascular risk/disease, understanding of the benefits, and accessibility including time and location.^17^

The patient–professional relationship and use of behavioural change techniques such as motivational interviewing can positively influence patients' motivation to make lifestyle changes.^22,23^ Previous research has identified the importance of HCPs interpreting and communicating results and information on risk, clearly and to ensure that it is personalized.^6,24,25^ Previous findings also reported that some patients sometimes found their QRisk2 score confusing and that it held no meaning or significance to them.^6,16,26^ There are potential limitations in relying on risk score as a trigger for facilitating behaviour change.^16^

In some instances, the results from an NHS Health Check provided reassurance, confirmed existing healthy lifestyle behaviours and triggered behaviour change.^6,21^ However, findings from other preventative programmes highlight the psychosocial barriers to modifying lifestyle behaviours such as preparedness for behaviour change, lack of confidence or self-efficacy.^21^ There are conflicting findings on the impact of attending preventative programmes.^21,22,25^ Some studies report that patients sometimes experience anxiety and stress associated with the receipt of negative or high-risk results^21,25^ whilst others found no associated harms from attending CVD checks.^22^

### What this study adds

Our findings suggest that many patients were motivated to attend an NHS Health Check due to health beliefs associated with the value of preventative healthcare. Patients valued the early detection and prevention of CVD risk, particularly when there was a family history of CVD. Other accounts reported in this study such as not wishing to be a burden on family or society may reflect the adoption or influence of prevailing discourses relating to the ‘burden’ of an ageing population.^27^ A few patients viewed the NHS Health Check as a general screening programme to identify non-CVD-related diseases.^6,17^ This highlights the importance of ensuring the purpose of the NHS Health Check is communicated clearly by practices and in associated publicity.

HCPs highlighted their concerns about inequity in uptake and evidence of the inverse care law^28^ in terms of low attendance from those who are most likely to benefit. This view has been supported in some quantitative literature associated with the uptake of an NHS Health Check.^12–14^ Previous research has demonstrated that those at highest risk of CVD are least likely to attend a Health Check.^29^ Targeting NHS Health Checks at patients identified as most likely to benefit from them may be more effective^30^ and avoid investing scarce resources in the worried well. Practices and commissioners may need to consider the expansion of NHS Health Checks into targeted community settings, e.g. workplace, community pharmacies, to increase community engagement to promote equitable uptake. Early evidence suggests that the uptake and acceptability of NHS Health Checks in community settings may offer a viable alternative.^16,31,32^

These findings from both professional and, notably, patient accounts are a reminder of the need to ensure HCPs are suitably equipped to interpret and communicate information accurately and sensitively. Suitable guidance and training needs to be provided to HCPs to ensure that high-risk results are followed-up swiftly and managed sensitively to avoid patients experiencing anxiety. In circumstances where patients are invited for follow-up tests, in response to high, medium or borderline risk results, HCPs need to provide reassurance and clear information about options, to avoid or alleviate anxiety amongst patients. As supported by recent evidence,^16,26^ some patients in this study had not understood the significance of CVD risk factors such as high blood pressure or high QRisk2 scores which may limit the value of attending.^16^ HCPs may also need to be aware of differences in patients' interpretation of their QRisk2 score; for example, a QRisk2 score of 20% may be interpreted differently by different patients and may influence subsequent decision-making processes concerning behavioural change. Additionally, previous evidence suggests that there are often discrepancies between patients' understanding of cardiovascular risk for example, associated with poor diet and their subsequent intention to make lifestyle modifications.^33^ Patients' decisions are also influenced by their social context and relationships.^33^ In accordance with NICE guidelines,^34^ HCPs need to ensure that information about risk is communicated both verbally and in written formats, adopting a patient-centred approach which takes account of individuals' understanding of risk, and their personal and social circumstances and preferences.

Some criticism was directed at HCAs by some GPs and nurses. This related to whether HCAs had the full complement of skills and knowledge required to deliver Health Checks meaningfully. However, HCAs stated that they felt confident in their role and perhaps the concerns raised by some nurses and doctors reflect an interest in maintaining professional boundaries. Additionally, concerns raised by patients about their anxiety associated with the receipt of unexpected results were attributable to consultations delivered by both nurses and HCAs. The findings from this study suggest that HCPs may be failing to provide personalized lifestyle advice, tailored to the individual in order to meaningfully inform, motivate and support patients to make lifestyle changes, as suggested in NHS Health Checks guidance.^34^ HCPs need to consider and acknowledge the psychosocial constraints which may influence a person's ability or preparedness to make changes. Adopting such approaches has also been highlighted in a recent draft competency framework for HCPs delivering NHS Health Checks.^35^ This emphasizes the need to ensure HCPs employ behaviour change techniques such as motivational interviewing ‘to deliver patient appropriate lifestyle advice, and how this can contribute towards reducing their risk of CVD’ (Ref. 35, p. 20). Public Health England^5^ requires NHS Health Check commissioners to provide high-quality training to HCPs conducting NHS Health Checks and needs to consider ways of meeting the above training needs to ensure HCPs are competent to carry out health checks and support patients to make lifestyle changes.

Accounts from patients and HCPs have highlighted the implications of attending a health check, with mixed reactions received from patients. This relates to benefits from attendance, as reported previously, including relief and reinforcement of healthy lifestyles and lifestyle change in some.^6,21^ However, for some, anxiety may be experienced in relation to unexpected results and whilst awaiting re-tests. The emotional impact associated with NHS Health Checks in terms of their potential to raise anxiety is highlighted as a potential drawback in the general screening literature yet has not been widely reported in relation to NHS Health Checks.

### Limitations

Although the sample encompassed a range of patients in relation to age, SES and QRisk2 scores, it was drawn from a limited geographical area and most patients were of white British ethnicity, so it is possible that the views and experiences of patients from other locations or ethnicities would differ. Our findings are based on the accounts of a self-selected group of participants. The motivations and willingness of these participants to take part in a study may have implications for the generalizability of these findings. This study only reports the findings of patients who attended an NHS Health Check and it would therefore be valuable if future research could explore the perceptions of patients who did not attend. All interviews were conducted by the same researcher (R.R.), an experienced female health service researcher with a background in psychology, health promotion and medical sociology. The researcher's background may have influenced her interest in exploring participants' experience of the NHS Health Checks process from a more critical perspective, including an exploration of the potential benefits and disadvantages from both the patients' and professionals' perspective. The multidisciplinary team brought a range of perspectives on the interpretation and applicability of the findings.

## Conclusions

This study has identified the varied motivations for attending an NHS Health Check and the potential benefits and challenges. To improve patient satisfaction and improve the facilitation of lifestyle change, it is vital that HCPs conducting the NHS Health Checks receive the appropriate training to equip them with the key skills and knowledge to deliver the service using a patient-centred approach. To address concerns about equitability in uptake, the findings highlight the need to ensure Health Check commissioners provide alternatives to practice-based health checks and engage with communities who may be less likely to attend.

## Author's contributions

A.M. and J.H. were responsible for developing the research questions and study design; R.R. and J.H. for study management and data analysis; R.R., N.C., A.M., G.F. and J.H. writing the manuscript; R.R. accepted the final version. All authors read and approved the final manuscript.

## Funding

This study is funded by the National Institute for Health Research (NIHR) School for Primary Care Research (SPCR). This article presents independent research funded by the National Institute for Health Research (NIHR). The views expressed are those of the authors and not necessarily those of the NHS, the NIHR or the Department of Health.
